# Dynamics of cholera epidemics from Benin to Mauritania

**DOI:** 10.1371/journal.pntd.0006379

**Published:** 2018-04-09

**Authors:** Sandra Moore, Anthony Zunuo Dongdem, David Opare, Paul Cottavoz, Maria Fookes, Adodo Yao Sadji, Emmanuel Dzotsi, Michael Dogbe, Fakhri Jeddi, Bawimodom Bidjada, Martine Piarroux, Ouyi Tante Valentin, Clément Kakaï Glèlè, Stanislas Rebaudet, Amy Gassama Sow, Guillaume Constantin de Magny, Lamine Koivogui, Jessica Dunoyer, Francois Bellet, Eric Garnotel, Nicholas Thomson, Renaud Piarroux

**Affiliations:** 1 Department of Parasitology, Aix-Marseille University/UMR MD3, Marseille, France; 2 Department of Epidemiology and Biostatistics, University of Health and Allied Health Sciences, Ho, Ghana; 3 National Public Health and Reference Laboratory, Ghana Health Service, Accra, Ghana; 4 Regional Office for West and Central Africa, UNICEF, Dakar, Senegal; 5 Pathogen Genomics, Wellcome Trust Sanger Institute, Hinxton, Cambridge, United Kingdom; 6 Department of Bacteriology, National Institute of Hygiene, Lomé, Togo; 7 Department of Disease Surveillance, Ghana Health Service, Accra, Ghana; 8 Ministry of Local Government & Rural Development, Environmental Health and Sanitation Directorate, Accra, Ghana; 9 Sorbonne Université, INSERM, Institut Pierre-Louis d’Epidémiologie et de Santé Publique, Paris, France; 10 Department of Disease Surveillance, Ministry of Health, Lomé, Togo; 11 Department of Epidemiology and Health Surveillance of Borders, Ministry of Health, Cotonou, Benin; 12 Department of Bacteriology, Pasteur Institute, Dakar, Senegal; 13 UMR IRD224-CNRS5290-UM MIVEGEC, Institut de Recherche pour le Développement (IRD), Montpellier, France; 14 National Institute of Public Health, Ministry of Health, Conakry, Republic of Guinea; 15 Department of Bacteriology, Hôpital d'Instruction des Armées Laveran, Marseille, France; 16 Department of Pathogen Molecular Biology, London School of Hygiene & Tropical Medicine, London, United Kingdom; 17 Sorbonne Université, INSERM, Institut Pierre-Louis d’Epidémiologie et de Santé Publique, AP-HP, Hôpital Pitié-Salpêtrière, Paris, France; Vienna, AUSTRIA

## Abstract

**Background:**

The countries of West Africa are largely portrayed as cholera endemic, although the dynamics of outbreaks in this region of Africa remain largely unclear.

**Methodology/Principal findings:**

To understand the dynamics of cholera in a major portion of West Africa, we analyzed cholera epidemics from 2009 to 2015 from Benin to Mauritania. We conducted a series of field visits as well as multilocus variable tandem repeat analysis and whole-genome sequencing analysis of *V*. cholerae isolates throughout the study region. During this period, Ghana accounted for 52% of the reported cases in the entire study region (coastal countries from Benin to Mauritania). From 2009 to 2015, we found that one major wave of cholera outbreaks spread from Accra in 2011 northwestward to Sierra Leone and Guinea in 2012. Molecular epidemiology analysis confirmed that the 2011 Ghanaian isolates were related to those that seeded the 2012 epidemics in Guinea and Sierra Leone. Interestingly, we found that many countries deemed “cholera endemic” actually suffered very few outbreaks, with multi-year lulls.

**Conclusions/Significance:**

This study provides the first cohesive vision of the dynamics of cholera epidemics in a major portion of West Africa. This epidemiological overview shows that from 2009 to 2015, at least 54% of reported cases concerned populations living in the three urban areas of Accra, Freetown, and Conakry. These findings may serve as a guide to better target cholera prevention and control efforts in the identified cholera hotspots in West Africa.

## Author summary

We analyzed cholera epidemics from Benin to Mauritania, during 2009 to 2015, and performed a series of field visits as well as molecular epidemiology analyses of *V*. *cholerae* isolates from most recent epidemics throughout West Africa. We found that at least 54% of cases concerned populations living in the three urban areas of Accra, Freetown, and Conakry. Accra, Ghana represented the main cholera hotspot in the entire study region. Our findings indicate that the water network system in Accra may play a role in the rapid diffusion of cholera throughout the city. As observed in Accra, Conakry, and Freetown, once cholera cases arrive in overpopulated urban settings with poor sanitation, increased rainfall facilitated the contamination of unprotected water sources with human waste from cholera patients, thus promoting a rapid increase in cholera incidence. To more efficiently and effectively combat cholera in West Africa, these findings may serve as a guide to better target cholera prevention and control interventions.

## Introduction

Seven cholera pandemics have been documented since 1817 [1]. The disease has plagued every continent, spreading along trade routes via both land and sea [1]. Current epidemics are however localized to South Asia, Haiti, and Sub-Saharan Africa [2]. Since the ongoing pandemic first reached West Africa in 1970 [1], outbreaks have been repeatedly reported throughout the region [2]. However, only a few small-scale studies have investigated the dynamics of recent cholera epidemics in West Africa. Overall, cholera outbreaks throughout the study region have displayed markedly diverse patterns depending on the country. Certain countries such as Benin and Togo have reported cholera cases nearly every year albeit with relatively low incidence [3], [4]. By contrast, many other countries to the northwest such as Gambia, Senegal, and Mauritania have experienced marked multi-year lulls [5–7]. Many large epidemics erupted on the heels of violent civil conflicts that engendered humanitarian and public health crisis or massive population movement such as that in Senegal in 2004–2006 [7]. The large majority of cases were also reported from large cities following increased rainfall [7–10]. Although many studies were limited to a single outbreak/neighborhood or a short time period, common risk factors were found across the region: crowded living conditions, poor sanitation, and limited access to potable drinking water [3], [8–11].

Cholera is contracted by consuming food or water contaminated with toxigenic *Vibrio cholerae* O1 or the derivative *Vibrio cholerae* O139 [12]. Numerous *V*. *cholerae* non-O1 and some O1 serogroups lacking the cholera toxin are autochthonous in seawaters worldwide [13]. *V*. *cholerae* non-O1 serogroups have been found associated with a variety of aquatic flora and fauna, notably copepods [14]. In the Bay of Bengal, elevated seawater temperatures, copepod and plankton blooms, and rainfall have been shown to correlate with increased concentrations of *V*. *cholerae* in the environment [15–17]. In West Africa, a study has addressed the relationship between climate inter-annual variability and cholera in Nigeria, Benin, Togo, Ghana, and Ivory Coast over a 20-year period. From 1987–1994, they observed temporospatial synchrony between cholera incidence and rainfall in all countries except Ivory Coast [18]. Cholera has thus been depicted as a waterborne disease driven by ecological factors [12], [19]. However, despite technological improvements, a perennial aquatic reservoir of cholera-causing *V*. *cholerae* O1 has yet to be identified in West Africa [20].

We applied an integrated approach to describe the dynamics of cholera epidemics, including the identification of hotspots, and investigate factors that may influence the disease in several coastal West African countries from Benin to Mauritania. We analyzed weekly cholera outbreak evolution (at the district or commune level) throughout the region between 2009 and 2015 and field investigations in Benin, Togo, Ghana, Ivory Coast, Sierra Leone, and Guinea. We performed molecular epidemiology analysis (MLVA (Multi-Locus VNTR [Variable Number Tandem Repeat] Analysis) and whole-genome sequencing) of *V*. *cholerae* isolates from the majority of outbreaks affecting the study region since 2010. Such molecular epidemiology analysis can supplement epidemiological findings to provide further insight into the relationship between *V*. *cholerae* isolates and epidemic populations, identify clusters, establish phylogeny, and track bacterial transmission. We describe our findings country by country, from Benin to Mauritania.

## Methods

### Cholera case and rainfall data

Databases of all suspected cholera cases were collected from the epidemiological units of Benin, Togo, Ghana, and Guinea. National databases comprised weekly case/death numbers at the district level (Ghana and Togo), commune level (Benin), or prefecture level (Guinea) since 2009, according to the WHO cholera case definition (S1 Text). For Ghana (Accra), Togo (Lomé), Benin (Atlantique and Littoral/Cotonou), and Guinea (Conakry), we also analyzed the cholera case line lists, which include data on age, sex, clinical outcome, and residence. We obtained approval from the Ministry of Health (MoH) of each country to use these databases for epidemiological, research, and publication purposes. The line lists were anonymized and cleaned prior to analysis.

Daily-accumulated rainfall data for the Greater Accra Region (GAR) were obtained from satellite estimates (TRMM_3B42RT_DAILY.007) from NASA (http://disc.gsfc.nasa.gov/precipitation/tovas).

### Field visits

For each field visit carried out in Ivory Coast, Ghana, Togo, and Benin, we first organized and coordinated the study with UNICEF and WHO (for Ivory Coast), through whom we were put in contact with national authorities to obtain official access to databases and *V*. *cholerae* strains (when possible). Initial field visits involved contact with these local UNICEF and/or WHO offices as well as national public health (surveillance and laboratory) authorities. To identify cholera hotspots and sites that may play a role in cholera diffusion, we first analyzed outbreak histograms and mapped weekly cholera cases. Field visits were then performed in the identified locales (e.g., sites that were repeatedly affected by cholera outbreaks or sites of an initial outbreak). During field visits, and accompanied by local counterparts, we met with local surveillance departments, laboratories (for information concerning lab-based case confirmation), and health facilities. Information was collected concerning how cholera was contracted as well as possible links with other cases. We also evaluated local WASH (Water, Sanitation, and Hygiene) conditions.

Field visits were performed in Ivory Coast (12/2013; SM and RP); Ghana, Togo and Benin (11-12/2014; SM, PC, and RP); Guinea and Sierra Leone (08-09/2012; SR). See S1 Text for additional details concerning the field visit study protocol.

### Ethics

The study was approved by the MoH of each country where field visits were carried out. The protocol was further approved by the Ghana Health Service Ethical Review Board according to standard procedures. The remaining countries did not seek ethics approval as epidemic disease surveillance and response is covered by national public health laws as an integral part of the public health mandate of each MoH.

All clinical isolates studied were analyzed anonymously.

### Cartography

The country maps were generated using QGIS v2·8-Wien with shapefiles from DIVA-GIS (http://www.diva-gis.org/gdata). The shapefile of Accra was generated with QGIS in collaboration with the Ministry of Local Government, Division of Environmental Health, Accra.

### *Vibrio cholerae* isolate culture, DNA isolation, MLVA, and whole-genome sequencing

To design effective public health strategies to combat cholera, it is critical to understand the mechanisms of cholera emergence and diffusion in a region-specific manner. Genetic analysis of responsible strains can supplement epidemiological findings to provide further insight into the relationship between pathogenic strains and epidemic populations [21]. Indeed, isolate genotyping is useful to differentiate between different isolates, identify clusters, establish phylogeny, and track bacterial transmission. Lam et al. [22] have shown that MLVA represents a highly discriminatory technique to distinguish between closely related seventh pandemic isolates. They have also emphasized that the method is best applied for outbreak investigations or to identify the source of an outbreak. Rebaudet et al. have recently demonstrated that MLVA-based analysis of clinical *V*. *cholerae* isolates combined with an epidemiological assessment was instrumental in deciphering the origin of the 2012 cholera epidemic in Guinea [10].

All *V*. *cholerae* isolates were selected in a manner as to represent both the spatial and temporal evolution of each cholera epidemic in each country sampled. A total of 173 *V*. *cholerae* O1 clinical isolates collected throughout Ghana from 2010 to 2014 were provided by the National Public Health Reference Laboratory, Accra. The isolates were sub-cultured and transported in glycerol tubes at ambient temperature to Marseille, France. Aliquots of the culture were directly submitted for DNA extraction. We also analyzed three *V*. *cholerae* isolates from Senegal in 2011 following the same procedure. The MLVA results of the Ghanaian isolates were compared with those of previously analyzed strains from Togo (35 isolates), Guinea (37 isolates), and Sierra Leone (9 isolates) as previously described [10], [23].

DNA was extracted using a NucliSENS easyMAG platform (bioMérieux). MLVA of the isolates using an ABI PRISM 3130 Genetic Analyzer (Applied Biosystems) was performed using six VNTRs as described previously [23], and the relationship between the isolates was established using the goeBURST algorithm on PHYLOViZ v1.1 (http://www.phyloviz.net/). We performed a phylogenetic assessment of the core *V*. *cholerae* genome of the strains of the third wave of the seventh pandemic [24] based on genome-wide SNPs. Isolates from Togo-2010 (six), Togo-2011 (six), Togo-2012 (five), Ghana-2011 (one), Ghana-2014 (five), and Guinea-2012 (two) were also included in the analysis. DNA was sequenced using a HiSeq Illumina System (Illumina) and analyzed as described [24].

## Results

### Cholera epidemics from Benin to Mauritania

From 2009 to 2015, Benin and Togo accounted for a combined average of 694 reported cholera cases annually (Table 1). In Benin, the lakeside commune of Sô-Ava, which is directly connected to Nigeria via Lake Nokoué and Yewa River, reported cholera outbreaks every year since 2010 and was often the first and hardest-hit commune. In Benin, Sô-Ava reported 40% of all cases in 2013 and 30.4% of all cases in 2014 (S1 Fig). Cotonou, the economic center of Benin, was only affected by limited cholera outbreaks in 2010, 2011, and 2013, in neighborhoods characterized by fishing activity and pronounced population movement (S1 Fig). In neighboring Togo, most outbreaks in Lomé, the capital of Togo, occurred in flood zones (Lomé D2: Adakpamè, Bè Kpota, Anfamé, and Akodéssewa) or areas linked with fishing activity and intense population movement (Lomé D3: Katanga) (S2 Fig). Meanwhile, the outbreaks in the eastern Lacs Prefecture, Togo (in Séko) were associated with people attending traditional animist ceremonies, including those who traveled from Benin or Nigeria, as noted by health facility staff in Séko. Outbreaks in Togo often remain limited (S2 Fig).

**Table 1:** The number of suspected cholera cases reported in each country included in the study per year.

Country	Suspected cholera cases reported							
	2009	2010	2011	2012	2013	2014	2015	Total
**Coastal West African countries included in the epidemiological study**								
**Ivory Coast**	5	32	1261	424	56	235	199	2212
**Guinea**	42	0	3	7350	319	1	0	7715
**Benin**	74	983	775	668	528	832	0	3860
**Togo**	218	72	33	61	194	262	35	875
**Liberia**	1070	1546	1146	219	92	44	0	4117
**Sierra Leone**	0	0	0	23124	377	0	0	23501
**Ghana**	1294	438	10387	9563	20	28944	692	51338
**Guinea-Bissau**	5	0	0	3068	969	11	0	4053
**Senegal**	4	3	5	1	0	0	0	13
**Mauritania**	0	0	46	0	0	0	0	46
**The Gambia**	0	0	0	0	0	0	0	0
**Countries neighboring the study region**								
**Burkina Faso**	0	0	20	143	0	0	0	163
**Nigeria**	13691	44456	23377	597	6600	35996	5290	130007
**Niger**	0	1154	2324	5284	585	2059	51	11457
**Mali**	0	0	2220	219	23	0	0	2462

Reported cases in the countries neighboring the study region are also indicated. The data is based on Weekly Epidemiological Records [2].

**Fig 1:**
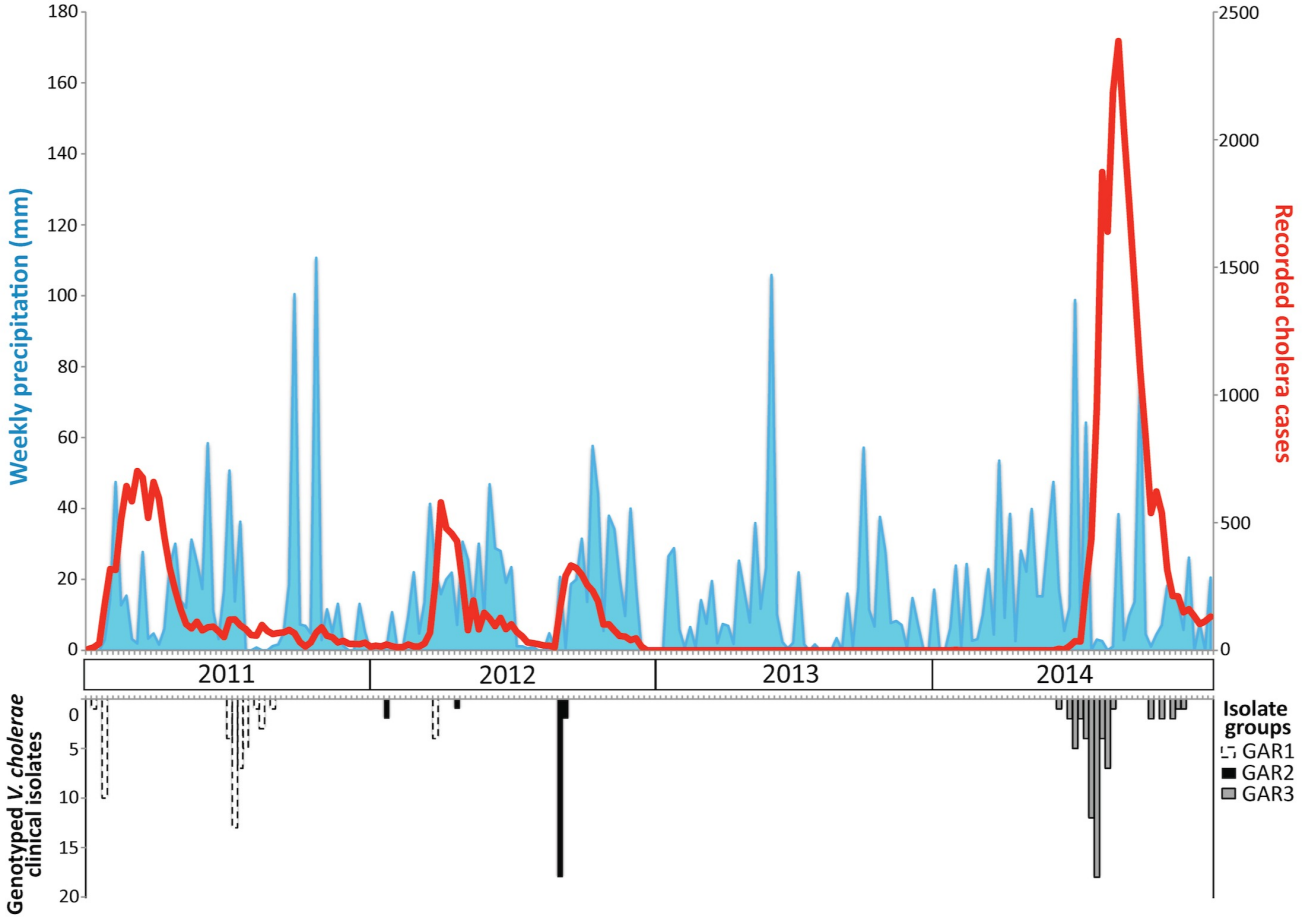
Weekly evolution of cholera epidemics, rainfall levels, and the tested *V*. *cholerae* isolates in Greater Accra Region from 2011 to 2014. Suspected cholera cases are indicated in red (right y-axis), and rainfall is indicated in blue (left y-axis). The corresponding year is labeled on the x-axis. To integrate the epidemiological and MLVA/MST data, the three major MST clusters identified in Accra are indicated below the histogram of suspected cholera cases. GAR1 = the Ghana 2011 cluster (which gave rise to a few strains in 2012), GAR2 = the main Ghana 2012 cluster, and GAR3 = the Ghana 2014 cluster identified on the MST.

Ghana accounted for 52.4% of all reported cholera cases in coastal countries from Benin to Mauritania, from 2009 to 2015 (51,333 suspected cases in Ghana / 97,887 total suspected cases in the 11 countries) [2]. Since 2011, cholera outbreaks have significantly intensified in Accra, the capital of Ghana. The majority (73.6%) of cases from 2011 to 2014 were reported in the Greater Accra Region (GAR) (35,985 cases GAR/ 48,914 cases Ghana) (MoH). In 2012 and 2014, the first confirmed cholera cases were detected in GAR. By contrast, the 2011 epidemic began in late 2010, during which the first detected cases were in Central Region (S3 Fig). The 2010/2011 epidemic then intensified in GAR in January 2011, as displayed in Fig 1. Fig 1 displays the sharp increase in cases at the onset of each epidemic, indicating a rapid early expansion of the bacterium within Accra. Strikingly, from the end of 2012 through mid-2014, Ghana experienced an 18-month lull in cholera cases, despite typical rainfall. All 20 suspected cholera case samples in 2013 tested negative for *V*. *cholerae*. After this significant lull, Ghana experienced the largest epidemic (28,944 cases in 2014) since 1991 [2]. Notably, the strains causing this large outbreak in 2014 were closely related with strains present in Togo in 2010 and 2011, as demonstrated by the MLVA and phylogeny data described in further detail below.

**Fig 2:**
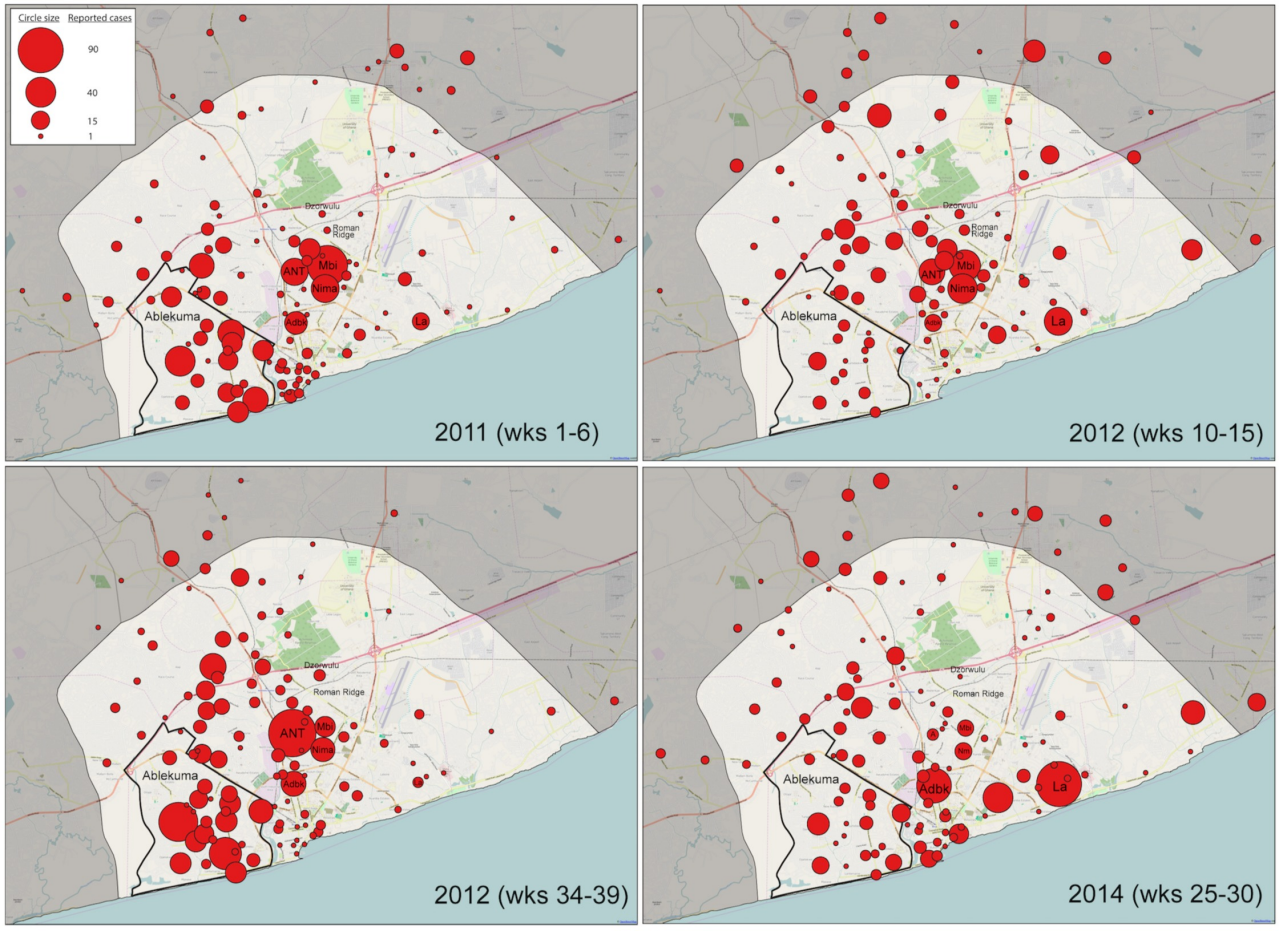
Distribution of cholera cases during the initial six weeks of epidemic escalation in Accra Metropolis from 2011 to 2014. The cumulative cases reported in the Accra Metropolis line list for each neighborhood during the first six weeks of each epidemic are indicated with red circles. Circle size represents the relative number of cases reported in each neighborhood. The neighborhoods of Maamobi, Nima, Accra New Town, and Adabraka often reported many cases during the first six weeks of each outbreak. Many parts of Ablekuma reported several cholera cases during outbreak onset. Labadi was the hardest-hit zone during the beginning of the 2014 epidemic. In contrast, certain nearby residential areas remained largely cholera-free (e.g., Dzorwulu and Roman Ridge), despite major outbreaks in adjacent neighborhoods. The total cases accounted for and percentage coverage of sites localized for each epidemic are as follows: 2011 (897 cases; 88%), first 2012 epidemic (780 cases; 92%), second 2012 epidemic (932 cases; 94%), and 2014 (913 cases; 86%). The districts adjacent to Accra Metropolis are grayed out. Abbreviations: ANT/A, Accra New Town; Mbi, Maamobi; Adbk, Adabraka; La, Labadi; Nm, Nima.

Once outbreaks erupted in Accra, cholera rapidly diffused throughout the majority of the city. This rapid spatial diffusion pattern was observed during the onset of the 2011, 2012, and 2014 epidemics. Many Accra neighborhoods were severely affected by cholera each year. However, certain nearby residential areas remained largely cholera-free, despite outbreaks in adjacent neighborhoods (Fig 2). Once cholera erupted in Accra, outbreaks spread to other districts in Ghana several weeks later. According to health facility staff at the hospital in Ho (Volta Region), the 2014 index case in Ho had recently traveled from Accra, where an outbreak was ongoing at the time (S4 Fig).

Field visits revealed that water distribution was often interrupted for several days in many Accra neighborhoods. The majority of water network pipes were visibly damaged and running along the ground through roadside gutters. Residents without access to proper latrine facilities perform open defecation into these gutters. In the Greater Accra Metro Area, access to improved sanitation facilities is limited. Only 5.7% of households in Greater Accra have access to latrines that flush into a piped sewer system, while 35.1% of Accra households use latrines that flush into a septic tank. Furthermore, Accra households lacking improved sanitation facilities (14.8%) are forced to use pan latrines (buckets that are then dumped into the roadside gutters) or “flying toilets” (defecation into a plastic bag which is then literally thrown away) [25]. We thus hypothesize that ground water and human waste could seep into broken pipes, especially during the frequent water shortages, thereby allowing *V*. *cholerae* to enter the water network and spread when water pressure is restored. Increased rainfall markedly exacerbated this effect (Fig 1 and Fig 2). Additional studies should further investigate the role that the Accra water network plays in cholera outbreaks to confirm this hypothesis.

From 2009 to 2010, Ivory Coast was largely unaffected by cholera (37 suspected cases) [2]. However, an epidemic erupted in Abidjan in January 2011 following the post-election crisis of November 2010 and a collapse in the health and sanitation systems [26]. The 2011 epidemic was responsible for 1,261 cases [2]. We found that a new outbreak emerged in May 2012 in Sud Comoé, adjacent to Jomoro District in Ghana, where an outbreak was ongoing. As observed in Ghana, Ivory Coast also experienced a complete lull in cholera in 2013 and early 2014. Each of the 56 suspected cases in 2013 tested negative for *V*. *cholerae* [27]. This lull was interrupted in early October 2014, when an outbreak occurred in Abidjan with the arrival of ill Ghanaian fishermen (S5 Fig) [28].

Liberia has reported 4,133 suspected cholera cases from 2009 to 2015 [2], which includes only 44 cases in 2014 and zero cases in 2015. However, no cholera-related deaths were reported from 2010 to 2013, and only two deaths (1,070 cases) were reported in 2009.

Following a three-year lull in the incidence of cholera, both Sierra Leone and Guinea experienced a trans-border epidemic in 2012, with 22,932 and 7,351 cases, respectively [10]. The two epidemics progressed following a very similar pattern. In Guinea, the outbreak started in February on Kaback Island with a fisherman traveling from Sierra Leone. In Sierra Leone, possible events of *V*. *cholerae* importation by fishermen travelling from Liberia and Ghana have been reported [29]. During the rainy season, cholera exploded in the capitals, which recorded over half of the total cases (Freetown, 52%; Conakry, 64%) [10], [29].

As cholera rates declined in Sierra Leone and Guinea, they rose in Guinea-Bissau [29–31], which also followed a near three-year lull. The country reported 3,068 cases in 2012 and 969 cases in 2013. Eighteen and zero cases were reported in 2014 and 2015, respectively [2].

The number of cholera cases reported in The Gambia, Senegal, and Mauritania has been very low since 2009 to present. The Gambia has not reported a single suspected cholera case since 2008. Likewise, Mauritania has not reported cholera cases since 2008, with the exception of 46 cases in 2011. Since 2009, Senegal has reported only 13 suspected cholera cases [2].

### Genetic analyses of *V*. *cholerae* isolates

**Fig 3:**
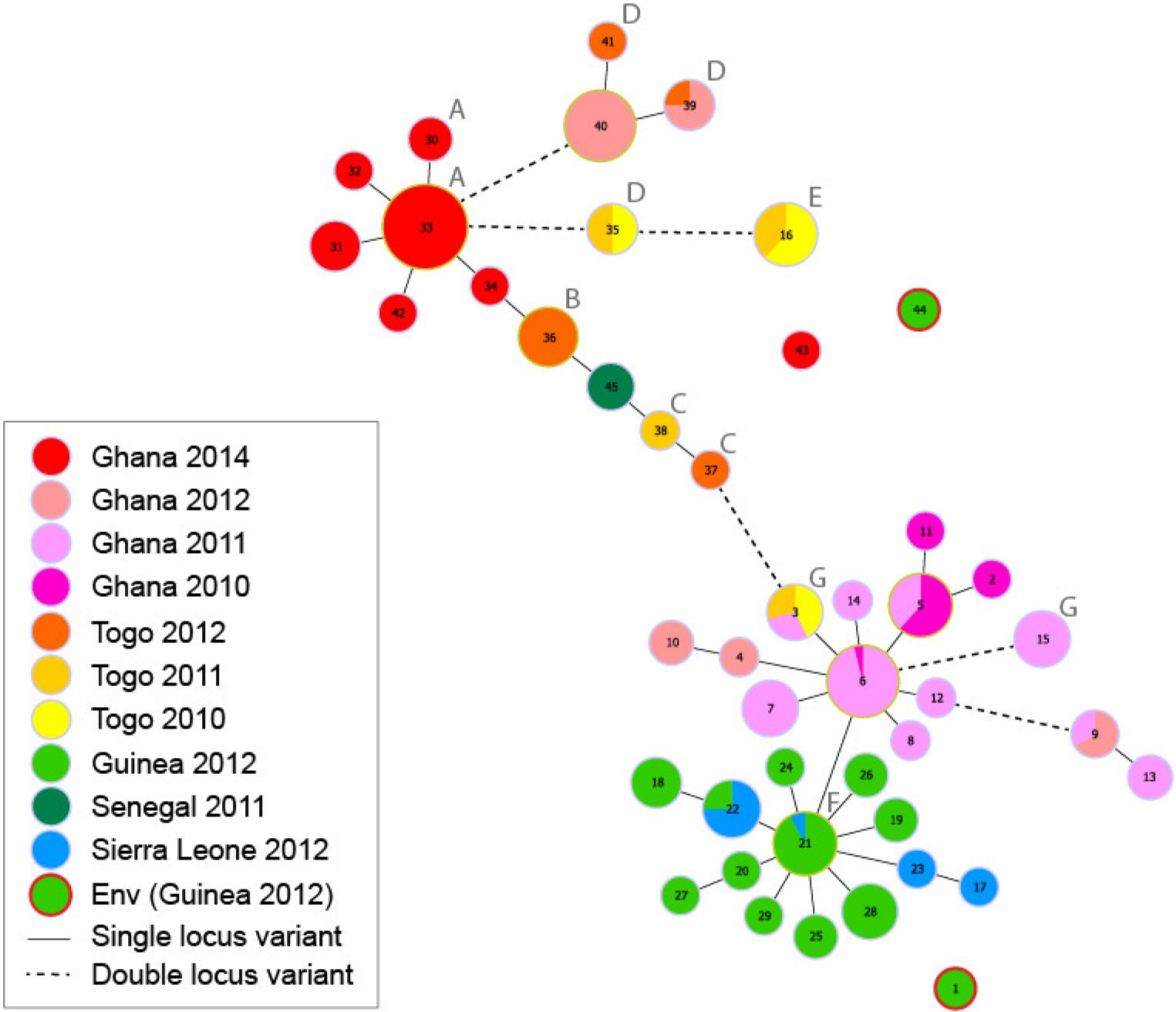
Minimum Spanning Tree based on the MLVA types of 257 *V*. *cholerae* isolates from several recent West African cholera outbreaks. Each MLVA type is represented by a node (and a unique number), and the size of the nodes reflects the number of isolates of each MLVA type. The solid lines indicate the most likely single locus variant, while dashed lines indicate the most likely double locus variant. The colors reflect the distinct country and year of isolate origin. Pie charts indicate strains from different time periods or countries displaying an identical MLVA type. The two strains represented by MLVA types #1 and #44 were isolated from environmental samples in Guinea (encircled in red). Labels A through G indicate the isolates from Ghana, Togo, and Guinea included on the phylogenic tree in Fig 4.

We performed MLVA of 255 clinical *V*. *cholerae* isolates from Ghana, Togo, Guinea, Sierra Leone, and Senegal. Two environmental isolates from Guinea 2012 were also included (S1 Table). Interestingly, the Minimum Spanning Tree (MST) shows that the 2010/2011 isolates from Ghana were related to those that seeded the epidemic in Guinea and Sierra Leone in 2012. The MST also demonstrates that the 2011, 2012, and 2014 epidemics in Ghana were due to three distinct *V*. *cholerae* MLVA-type clusters. The three clinical isolates from Senegal in 2011 displayed an identical MLVA type, closely related to strains from Togo in 2011 and 2012, which may represent imported cases from farther south in West Africa (Fig 3).

**Fig 4:**
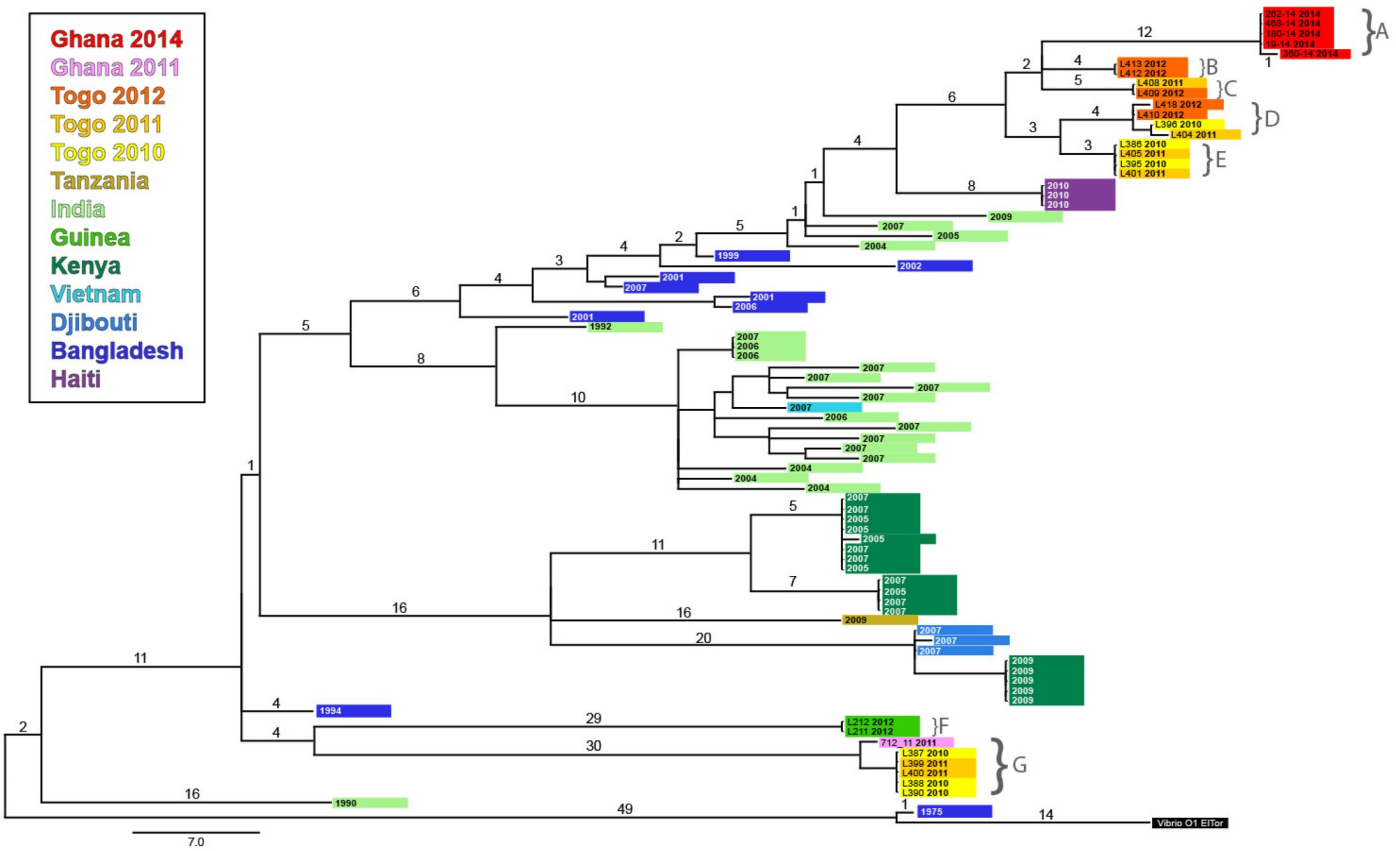
Strains from Ghana, Togo, and Guinea situated on the maximum likelihood phylogenetic tree of the third wave of the seventh pandemic lineage of *V*. *cholerae*. The tree is based on the SNP differences across the whole core genome. An isolate from the first wave, Bangladesh 1975, was included as an outgroup to root the tree. An isolate from the second wave was also included (India 1990). The color of the branch tips indicates the country of origin, and the year of isolation is specified. The strains from Ghana, Togo, and Guinea are indicated using the same colors as in the Minimum Spanning Tree (Ghana in pink and red, Togo in orange and yellow, and Guinea in bright green). Labels A through G indicate the isolates from Ghana, Togo, and Guinea included on the MST in Fig 3. Scale is provided as the number of substitutions per variable site, and the SNPs are indicated on the branches.

When the Ghana and Togo strains were included in the alignment of the seventh pandemic *V*. *cholerae* isolates [24], the Ghana 2011 and closely linked strains from Togo (2010 and 2011) grouped with the Guinea 2012 strains on the third wave of the current pandemic. By contrast, Ghana 2014 and other Togo strains clustered together in a separate clade, genetically distinct from the Ghana 2011 cluster. Some of the strains from Togo in 2012 clustered together with Ghana strains from 2012, which were more closely related to the Ghana 2014 strains on the MST (Fig 4).

## Discussion

From 2009 to 2015, we found that Accra, the capital of Ghana, reported the highest number of cholera cases during the study period among all cities included in this study. During the period 2009 to 2015, we found that one major wave of cholera outbreaks spread from Accra in 2011 northwestward to Sierra Leone, Guinea, and likely Guinea-Bissau in 2012 (S5 Fig). Genetic analysis showed that the 2012 isolates from Guinea and Sierra Leone clustered with those collected in Ghana in 2011. MLVA also demonstrated that the *V*. *cholerae* strain responsible for the epidemic in Ghana, which started in 2010 and spilled over into 2011, was already present in Togo and thus likely shared a common ancestor with strains from Togo in 2010. The MST and whole-genome sequence results indicate the presence of different *V*. *cholerae* populations in Togo, one group that appears to give rise to the 2011 Ghana strains, while the other group was closely related to the strain that triggered the cholera epidemic in Ghana in 2014. We noted that other cities (Conakry and Freetown) also appeared to function as amplifiers of cholera, when cases were present and rainfall increased. Strikingly, we found that many countries deemed cholera endemic in modeling studies [32] actually suffered very few outbreaks, with multi-year lull periods during which no cholera cases were detected. Extended lulls in cholera incidence occurred despite increased rainfall, typical high temperatures, slums, and population exposure to coastal environments. According to the WHO, cholera endemic countries have been defined as countries in which confirmed cholera cases were reported in at least three of the five past years [32]. In fact, some of the countries in our study do not fit the definition of endemic, as at least one case must be confirmed. To improve the current classification methodology, we may consider repeated, lab-confirmed cholera outbreaks in a country to indicate endemicity, keeping in mind that this status is fluid and may change as sanitation and water conditions improve.

Our findings and independent reports indicate that the Accra water network may play a role in rapid diffusion of cholera throughout a majority of the city, when cholera cases are present in neighborhoods where sanitary facilities and access to safe water are lacking. A study in Accra (in Osu Klottey Sub Metro) has shown that drinking community pipe-borne water (OR = 2.15) was associated with cholera in 2012 [33]. Furthermore, a separate study has revealed unsuitable residual chlorine levels and the regular presence of fecal coliform in the Accra network water [34].

During the period 2009 to 2015, our findings show that one major wave of cholera epidemics spread northwestward from Accra in 2011 to Sierra Leone and Guinea in 2012. As Ghanaian strains from previous years fail to reappear during subsequent epidemics, we hypothesize that epidemics affecting Accra may likely originate due to imported cases from a nearby cholera hotspot. Neighboring Nigeria represents one of the major cholera foci in the world [35], with 130,007 cases reported from 2009 to 2015 [2]. The country is also a likely source of outbreaks in the Lake Chad Basin [35]. The lull in Ghana during 2013 paralleled a relatively low number of cholera cases reported in Nigeria in 2012 and 2013. The 2014 Ghanaian epidemic coincided with an epidemic rebound in Nigeria [2]. Furthermore, intense commercial activity via road and boat may represent a major pathway by which cholera is imported from outbreaks in Nigeria to susceptible waterfront communities in Benin. We hypothesize that the detection of closely related strains in Ghana and Togo throughout the study period indicates that this strain may share a common ancestor with strains responsible for persisting outbreaks in a neighboring cholera hotspot such as Nigeria.

A phylogenetic analysis of clinical isolates has shown that the current pandemic is characterized by successive global clonal expansion of three waves of closely related serotype O1 El Tor lineages emanating from the Bay of Bengal [24]. A recent study in Science by Weill et al. has analyzed genomic data from 1070 *V*. *cholerae* O1 isolates, across 45 African countries and over a 49-year period, to show that past epidemics were attributable to a single expanded lineage. Weill et al. show that seventh cholera pandemic *V*. *cholerae* El Tor sub-lineages from Asia were repeatedly introduced into West Africa as well as East and Southern Africa. Epidemic waves then propagated regionally over a period of several years, which correlates with our observations [36]. Their findings strongly suggest that human factors play a much more important role in cholera dynamics and the long-term spread and maintenance of *V*. *cholerae* in Africa than environmental factors [36]. Further whole-genome sequence analysis of strains circulating in West Africa would greatly enhance our understanding of *V*. *cholerae* transmission pathways in the region.

Concerning the study limitations, although the databases used in this study are reliable thanks to cholera surveillance standardization throughout the region (S1 Text), the system does not guarantee detection of every single sporadic or unique imported cholera case. However, cholera cases are quickly detected once an outbreak occurs, especially when cholera-related deaths result. Many countries deemed cholera endemic have experienced several year-long lulls in cholera outbreaks (when all suspected cholera cases are confirmed negative upon culture). Nevertheless, countries considered cholera endemic anticipate outbreaks and perform culture-based tests on samples derived from suspected cholera cases each year. Given the capacity of the countries concerned (which can vary over time and geographically within a country), it is not possible to bacteriologically confirm every suspected case, which could have an effect on the validity of the results. Similar issues concerning surveillance capacity may have affected the completeness of suspected cholera case data collection over time. Nevertheless, our investigations have found that the lack of cholera during a lull period is not due to inadequate surveillance or laboratory incompetence but simply due to the absence of cholera cases in the country. The nonexistence of cholera outbreaks during the recent Ebola crisis in Guinea, Sierra Leone, and Liberia highlights the complete lull in cholera cases despite a heightened disease surveillance system. Furthermore, all isolates analyzed via MLVA were not submitted for whole-genome sequencing due to inadequate shipping conditions, which yielded sufficient DNA for only PCR-based MLVA. *V*. *cholerae* isolates from countries such as Ivory Coast, Benin, Guinea-Bissau, and Liberia were not included as the concerned national laboratories did not provide isolates for this investigation. We also acknowledge that our study would be strengthened by sequence-based phylogenic results of all samples assessed via MLVA, and thus efforts are currently underway to complete our panel of *V*. *cholerae* isolates. Furthermore, we were not able to include all isolates for sequence analysis, as certain samples lacked sufficient concentrations of DNA for the technique, thus introducing a possible bias due to incomplete selection of samples for whole-genome sequencing.

To prevent expansion of cholera outbreaks in the analyzed region of West Africa, epidemiological surveillance should be enhanced in identified vulnerable zones, such as Accra. In vulnerable areas, improved monitoring of the drinking water supply as well as ensuring water quality and proper chlorination would mitigate epidemics and perhaps stop cholera propagation.

Based on our findings, we hypothesize that once cases arrive in the urban settings with poor sanitation facilities (as observed in Accra, Conakry, and Freetown), increased rainfall provokes the infiltration of human waste, and therefore toxigenic *V*. *cholerae*, into the water network via damaged water pipes, thus promoting a rapid increase in cholera incidence. These findings may serve as a guide to better target cholera prevention and control interventions in the identified cholera hotspots in West Africa. Our study also highlights the value of this type of study combining epidemiological and molecular data to gain insight into the dynamics of cholera, especially in Africa.

## Supporting information

S1 Text Field visit details and protocol. (DOCX)

S1 Fig Weekly number of suspected cholera cases in Benin, by commune, from 2011 to 2014. The suspected cholera cases reported are indicated on the Y-axis and the weeks are indicated on the X-axis. The communes declaring cholera cases are indicated to the right of the corresponding histogram.
(TIF)

S2 Fig Weekly number of suspected cholera cases in Togo, by district, and in Lomé from 2013 to 2014. The top two histograms show weekly cholera cases by district in 2013 and 2014. The upper two histograms weekly cholera cases in Lomé in 2013 and 2014. Suspected cholera cases reported are indicated on the Y-axis and the weeks are indicated on the X-axis. The districts (upper histograms) and arrondissements of Lomé (lower histograms) declaring cholera cases are indicated to the right of the corresponding histogram. In 2012 only 61 cases were reported in Togo: 49 cases were reported in Lacs, 9 cholera cases came from Lomé (7 of which came from D2), and 3 cases were reported in Golfe. Only four cholera cases were reported in Togo in 2011.
(TIF)

S3 Fig Evolution of cholera cases in Ghana in 2010. Weekly cholera cases reported in Eastern, Central, and Ashanti Region are shown. The suspected cholera cases reported are indicated on the Y-axis and the weeks are indicated on the X-axis.
(TIF)

S4 Fig Map of Africa indicating the localization of sites visited. A zoom on the Guinea/Sierra Leone region (blue), southern Ivory Coast (purple), southern Ghana (red), and southern Togo and Benin (orange) are shown. In the Ivory Coast box, Sud Comoé (Ivory Coast) is indicated in pink and Jomoro District (Ghana) is indicated in yellow. In the Ghana box, Volta Region is indicated in purple and GAR (Greater Accra Region) is indicated in orange. In the Togo/Benin box, Lacs is indicated in red and Golfe is indicated in green.
(TIF)

S5 Fig Annual cholera cases reported in the study region from 2009 to 2015 and major identified events involved in the westward wave of epidemics from 2011 to 2014. Key events and observations involved in cholera epidemics: (1) events of *V*. *cholerae* importation between Benin, Togo, and Ghana; (2) the 2011 cholera epidemic in Ivory Coast broke out following the post-election crisis and public health breakdown; (3) in Ivory Coast, 2012 and 2104 index cases had traveled from Ghana; and (4) a *V*. *cholerae* clone was imported to Guinea by a fisherman from Sierra Leone (index case of the 2012 epidemic in Guinea). Countries reporting less than 50 cases for the study period are indicated with an asterisk. Abbreviations: SL, Sierra Leone; Lib, Liberia.
(TIF)

S1 Table The epidemic populations, isolate IDs, and PCR amplicon size of each allele corresponding to each MLVA type. The number of isolates corresponding to each MLVA type is indicated on the right. The environmental isolates are indicated with an asterisk (MLVA type column).
(DOCX)

## References

[1] Echenberg M (2011) Africa in the Time of Cholera: A History of Pandemics from 1817 to the Present, New York, NY, USA, Cambridge University Press.

[2] Anon (n.d.) ‘World Health Organization. Cholera, number of reported cases (data by country)’. *Global Health Observatory Data Repository* [online] Available from: http://www.who.int/gho/epidemic_diseases/cholera/en/ (Accessed 25 February 2016)

[3] Gbary AR, Dossou JP, Sossou RA, Mongbo V and Massougbodji A (2011) ‘Epidemiologic and medico-clinical aspects of the cholera outbreak in the Littoral department of Benin in 2008’. Med. Trop. (Mars), 71(2), pp. 157–61. 21695873

[4] Landoh Dadja Essoya, Gessner Bradford D, Badziklou Kossi, Tamekloe Tsidi, et al (2013) ‘National surveillance data on the epidemiology of cholera in Togo.’ The Journal of infectious diseases, 208 Suppl(Suppl 1), pp. S115–9. [online] Available from: http://www.ncbi.nlm.nih.gov/pubmed/24101639 (Accessed 29 September 2014) 24101639 10.1093/infdis/jit244

[5] Aidara A, Koblavi S, Boye CS, Raphenon G, et al (1998) ‘Phenotypic and genotypic characterization of Vibrio cholerae isolates from a recent cholera outbreak in Senegal: comparison with isolates from Guinea-Bissau’. Am J Trop Med Hyg., 58(2), pp. 163–167. 9502599 10.4269/ajtmh.1998.58.163

[6] Roquet D, Diallo A, Kodio B, Daff BM, et al (1998) ‘The senegalese cholera epidemic of 1995 to 1996, an example of the geographic approach to health studies [Article in French]’. Sante, 8(6), pp. 421–428. 9917565

[7] Manga NM, Ndour CT, Diop SA, Dia NM, et al (2008) ‘[Cholera in Senegal from 2004 to 2006: lessons learned from successive outbreaks]. [Article in French]’. Med. Trop. (Mars), 68(6), pp. 589–592. 19639824

[8] Ekra KD, Attoh-Touré H, Bénié BV, Coulibaly D, et al (2009) ‘[Five years of cholera surveillance in Ivory Coast during social and political crisis, 2001 to 2005] [Article in French]’. Bull Soc Pathol Exot, 102(2), pp. 107–109. 19583033

[9] Wendo C (2003) ‘Cholera spreads in Liberia’. Lancet, 362(9388), p. 966. 10.1016/S0140-6736(03)14401-7 14513844

[10] Rebaudet Stanislas, Mengel, Martin A, Koivogui Lamine, Moore Sandra, et al (2014) ‘Deciphering the Origin of the 2012 Cholera Epidemic in Guinea by Integrating Epidemiological and Molecular Analyses’. PLoS neglected tropical diseases, 8(6), p. e2898 doi: 10.1371/journal.pntd.0002898 24901522 10.1371/journal.pntd.0002898 PMC4046952

[11] Palma Pedro Pablo, Baron Emanuel, Luquero Francisco J, Banga Cunhate Na, et al (2011) ‘Cholera Epidemic in Guinea-Bissau (2008): The Importance of “‘ Place ‘”‘., 6(5), pp. 1–8. 10.1371/journal.pone.0019005 PMC3087718 21572530

[12] Harris Jason B, Larocque Regina C, Qadri Firdausi, Ryan Edward T and Calderwood Stephen B (2012) ‘Cholera’. Lancet, 379, pp. 2466–2476. doi: 10.1016/S0140-6736(12)60436-X 22748592 10.1016/S0140-6736(12)60436-X PMC3761070

[13] Chowdhury MA, Miyoshi S, Yamanaka H and Shinoda S (1992) ‘Ecology and distribution of toxigenic Vibrio cholerae in aquatic environments of a temperate region’. Microbios, 72(292–293), pp. 203–13. 1488021

[14] Vezzulli Luigi, Pruzzo Carla, Huq Anwar and Colwell Rita R (2010) ‘Environmental reservoirs of Vibrio cholerae and their role in cholera’. Environmental microbiology reports, 2(1), pp. 27–33. doi: 10.1111/j.1758-2229.2009.00128.x 23765995 10.1111/j.1758-2229.2009.00128.x

[15] Huq Anwarul, West Paul A, Small Eugene B, Huq M Imdadul and Colwell Rita R (1984) ‘Influence of Water Temperature, Salinity, and pH on Survival and Growth of Toxigenic Vibrio cholerae Serovar 01 Associated with Live Copepods in Laboratory Microcosms’. APPLIED AND ENVIRONMENTAL MICROBIOLOGY, 48(2), pp. 420–424. 6486784 10.1128/aem.48.2.420-424.1984 PMC241529

[16] Huq Anwar, Sack R Bradley, Nizam Azhar, Longini Ira M, et al (2005) ‘Critical Factors Influencing the Occurrence of Vibrio cholerae in the Environment of Bangladesh’., 71(8), pp. 4645–4654. doi: 10.1128/AEM.71.8.4645-4654.2005 16085859 10.1128/AEM.71.8.4645-4654.2005 PMC1183289

[17] Koelle Katia, Rodo Xavier, Pascual Mercedes, Yunus Md. and Mostafa Golam (2005) ‘Refractory periods and climate forcing in cholera dynamics’. Nature, 436(7051), pp. 696–700. doi: 10.1038/nature03820 16079845 10.1038/nature03820

[18] Constantin de Magny Guillaume, Guegan J F, Petit M and Cazelles B (2007) ‘Regional-scale climate-variability synchrony of cholera epidemics in West Africa’. BMC Infectious Diseases, 7(20), pp. 1–9. [online] Available from: http://ovidsp.ovid.com/ovidweb.cgi?T=JS&CSC=Y&NEWS=N&PAGE=fulltext&D=med5&AN=17371602%5Cn http://www.ncbi.nlm.nih.gov/pmc/articles/PMC1839095/pdf/1471-2334-7-20.pdf 10.1186/1471-2334-7-20 PMC1839095 17371602

[19] Singleton F L, Attwell R, Jangi S and Colwell R R (1982) ‘Effects of Temperature and Salinity Growth Vibrio cholerae’. APPLIED AND ENVIRONMENTAL MICROBIOLOGY, 44(5), pp. 1047–1058. 6295276 10.1128/aem.44.5.1047-1058.1982 PMC242147

[20] Rebaudet Stanislas, Sudre Bertrand, Faucher Benoît and Piarroux Renaud (2013) ‘Cholera in coastal Africa: a systematic review of its heterogeneous environmental determinants.’ The Journal of infectious diseases, 208 Suppl, pp. S98–S106. [online] Available from: http://www.ncbi.nlm.nih.gov/pubmed/24101653 (Accessed 14 November 2013) 24101653 10.1093/infdis/jit202

[21] Ramisse Vincent, Houssu Perrine, Hernandez Eric, Denoeud France, et al (2004) ‘Variable Number of Tandem Repeats in Salmonella enterica subsp. enterica for Typing Purposes’. Journal of clinical microbiology, 42(12), pp. 5722–5730. doi: 10.1128/JCM.42.12.5722-5730.2004 15583305 10.1128/JCM.42.12.5722-5730.2004 PMC535243

[22] Lam Connie, Octavia Sophie, Reeves Peter R and Lan Ruiting (2012) ‘Multi-locus variable number tandem repeat analysis of 7th pandemic Vibrio cholerae’. BMC Microbiology, 12(82), pp. 1–11. [online] Available from: BMC Microbiology 22624829 10.1186/1471-2180-12-82 PMC3438101

[23] Moore Sandra, Miwanda Berthe, Sadji Adodo Yao, Thefenne Hélène, et al (2015) ‘Relationship between Distinct African Cholera Epidemics Revealed via MLVA Haplotyping of 337 Vibrio cholerae Isolates.’ PLoS neglected tropical diseases, 9(6), p. e0003817 doi: 10.1371/journal.pntd.0003817 26110870 10.1371/journal.pntd.0003817 PMC4482140

[24] Mutreja Ankur, Kim Dong Wook, Thomson Nicholas R, Connor Thomas R, et al (2011) ‘Evidence for several waves of global transmission in the seventh cholera pandemic’. Nature, 477(7365), pp. 462–465. doi: 10.1038/nature10392 21866102 10.1038/nature10392 PMC3736323

[25] United Nations Children’s Fund, Institute of Statistical Social and Economic Research and University of Ghana (2014) *Multiple Indicator Cluster Survey*,

[26] World Health Organization: Global Task Force on Cholera Control (2011) *Cholera country profile: Cote d’Ivoire Cholera Outbreak in 2011*,

[27] World Health Organization (2014) *Coopération OMS—Côte d ‘ Ivoire 2012–2013 Rapport biennal*,

[28] United Nations (2014) *Cholera outbreak in the West and Central Africa: Regional Update, 2014 (WEEK 41)*,

[29] Dunoyer Jessica, Sudre Bertrand, Rebolledo Javiera and Cottavoz Paul (2013) ‘Le choléra transfrontalier en Sierra Leone et Guinée en 2012 et les stratégies d’intervention associées’. Action contre la faim—France, (2 2013), pp. 1–93. [online] Available from: https://www.humanitarianresponse.info/system/files/documents/files/RapportCholera_2013.pdf (Accessed 25 February 2016)

[30] Reliefweb (2012) ‘Guinea-Bissau: Cholera on the rise.’

[31] Anon (2013) ‘World Health Organization—CHOLERA COUNTRY PROFILE : GUINEA BISSAU’. Global Task Force on Cholera Control, (1), pp. 1–2. [online] Available from: http://www.who.int/cholera/countries/GuineaBissauCountryProfile2013.pdf?ua=1 (Accessed 25 February 2016)

[32] Ali Mohammad, Nelson Allyson R, Lopez Anna Lena and Sack David A (2015) ‘Updated Global Burden of Cholera in Endemic Countries’. PLoS neglected tropical diseases, 9(6), pp. 1–13. 10.1371/journal.pntd.0003832 PMC4455997 26043000

[33] Davies-Teye BBK, Vanotoo L, Yabani JB and Kwakye-Maclean C (2014) ‘Socio-Economic Factors Associated With Cholera Outbreak In Southern Ghana: A case-control study’. Value in Health, 17(3), p. A128.

[34] Karikari AY and Ampofo JA (2013) ‘Chlorine treatment effectiveness and physico-chemical and bacteriological characteristics of treated water supplies in distribution networks of Accra-Tema Metropolis, Ghana’. Appl Water Sci, 3, pp. 535–543.

[35] Piarroux R and Faucher B (2012) ‘Cholera epidemics in 2010: respective roles of environment, strain changes, and human-driven dissemination’. Clinical Microbiology and Infection, 18, pp. 231–238. doi: 10.1111/j.1469-0691.2012.03763.x 22288560 10.1111/j.1469-0691.2012.03763.x

[36] Weill François-xavier, Domman Daryl, Njamkepo Elisabeth, Tarr Cheryl, et al (2017) ‘Genomic history of the seventh pandemic of cholera in Africa’. Science, 358(11), pp. 785–789. doi: 10.1126/science.aad5901 29123067 10.1126/science.aad5901

