# Supplementary material for: Dynamics of cholera epidemics from Benin to Mauritania
Source: PLoS Negl Trop Dis. 2018 Apr 9;12(4):e0006379. doi: 10.1371/journal.pntd.0006379 (PMC5908202; doi:10.1371/journal.pntd.0006379)
Supplement: S1 Text — (DOCX) [file pntd.0006379.s001.docx]

**S1 Text. Detailed field investigation study protocol**

For each field investigation in Guinea, Côte-d’Ivoire, Ghana, Togo, and Benin, we first organized and coordinated the study with UNICEF (Guinea, Ghana, Togo, and Benin) or WHO (Côte-d’Ivoire). The organizing entity then put us in contact with national authorities to obtain official access to cholera databases and clinical *V. cholerae* strains (when possible). We also collected and analyzed all available reports and publications concerning cholera in each country visited. Once in the field, initial visits involved contact with these local UNICEF and/or WHO offices as well as national public health (surveillance and laboratory) authorities. Reports written by local investigators were collected when available.

Once cholera case databases were obtained, initial epidemiological analyses were conducted as follows:

- The databases were cleaned, organized and anonymized by an epidemiologist with the assistance of local public health experts
- We then perform a spatiotemporal analysis of cholera epidemics at incrementally finer administrative scales (e.g., national 🡪 district 🡪 city), sequentially zooming in on identified cholera foci
- These data were visualized spatially (via a series of sequential maps) and temporally (using case histograms)
- Outbreak characteristics were assessed, particularly with regard to time/place/person (age, sex, occupation), numbers, and place of residence

These initial epidemiological findings were used to determine the sites for field investigations: locales that were often affected by cholera outbreaks or sites of an initial outbreak (i.e., to understand the origin of the index case). During field investigations we visited with local surveillance departments, laboratories (for information concerning lab-based case confirmation), and health facilities. Information was collected concerning how cholera was contracted as well as possible links with other local cases. Interviews with local officials and health care staff were adapted to local conditions and situations.

The investigations conducted in Guinea, Sierra Leone, Ghana, Togo, and Benin were conducted during an epidemic, while a retrospective study was conducted in Cote d’Ivoire.

A team was not commissioned to investigate cholera in Mauritania, Senegal, and Gambia given the extremely low number of reported suspected cases for this study period. Concerning Senegal, the authors who live and work in the country confirmed the lack of clinical cases since 2011; they also helped us to contact laboratories and obtain the few clinical isolates collected during the study period. We did not investigate the details of the epidemic in Guinea-Bissau as the dynamics of cholera epidemics in this country has recently been published.^1^ Finally, we did not visit Liberia because the lethality associated with suspected cholera cases was extremely low in this country, which indicates that many suspect cholera cases notified by Liberia were likely not cholera. Furthermore, no cholera cases were detected in Liberia during the Ebola epidemic, at a time when many epidemiologists had strengthened disease surveillance and verified each suspicious death. Thus, we decided to focus on the countries most heavily burdened by the disease.

National case databases - sources and validity

This study was strengthened by the fact that cholera surveillance and outbreak response have recently been reinforced and harmonized in West Africa during workshops organized jointly by UNICEF and WHO.^2,3^ These workshops aimed to develop integrated and concerted strategies to control and prevent cholera and diarrheic diseases in the region. The aim was to standardize cholera surveillance throughout the region by (1) delineating a coherent definition of a suspected cholera case, (2) establishing a mandatory notification system (based on the WHO definition) of suspected cholera cases in each country, and (3) improving the response during outbreak onset. These efforts were also bolstered through specific programs implemented at the regional level by UNICEF, WHO, and other contributors (e.g., AFRICHOL consortium and Pasteur Institutes).

A similar process to confirm cholera outbreaks and data collection (organized and supervised at the national level) applied throughout the study region. In each country, the occurrence of suspected cholera cases (according to WHO case definition) triggers an investigation and the collection of stool specimens from patients meeting the case definition recommended by WHO.^4^ When rapid tests are available (e.g., in Benin), stool specimens are tested; if positive (=probable case), specimens (stool sample or rectal swab) are sent for cultures to the nearest available lab. If negative and suspicion remains high, stool samples are sent for cultures. All suspected cases, whether or not confirmed, are then recorded on line lists based on epidemiological links.

In Benin, Togo, Ghana, Côte-d’Ivoire, Liberia, Sierra Leone, Guinea, Gambia, Senegal and Mauritania a suspected cholera case was defined according to the WHO case definition:

- in an area where the disease is not known to be present, a patient aged five years or more develops severe dehydration or dies from acute watery diarrhea
- in an area where there is a cholera epidemic, a patient aged five years or more develops acute watery diarrhea, with or without vomiting
- a confirmed case is defined as any suspected case for whom the stool sample tests positive for *V. cholerae* O1

In Guinea-Bissau, during the 2012-2013 epidemic, a modified WHO clinical case definition was used for suspected and confirmed cases of cholera. A suspected case was defined as any person suffering from acute watery diarrhea with or without vomiting. A confirmed case was defined as any suspected case for whom the stool sample tests positive for *V. cholerae* O1 or O139.

**References**

1. Luquero FJ, Banga CN, Remartínez D, Palma PP, Baron E, Grais RF. Cholera epidemic in Guinea-Bissau (2008): the importance of "place". PLoS One. 2011 May 4;6(5):e19005. doi: 10.1371/journal.pone.0019005.
2. UNICEF/WHO/OCHA. Atelier de Dakar Lutte contre le choléra et les maladies diarrhéiques en Afrique de l’Ouest et du Centre. Dakar, Senegal, May 14-16, 2008. Available at: (<http://www.unicef.org/wcaro/04.Recommandations_Atelier_Dakar_FR_final__2009_06_19.pdf)> Accessed October 15, 2016.
3. Harmonization for Health in Africa/HHA. Rapport de l’Atelier technique HHA sur le choléra en Afrique de l’ouest et du centre – Final report. Dakar, Senegal, October 2-4, 2013. Available at: (<https://www.humanitarianresponse.info/system/files/documents/files/RapportAtelierTechniqueHHA_oct2013.pdf)> Accessed October 15, 2016.
4. Global Task Force on Cholera Control (2010) Cholera outbreak: assessing the outbreak response and improving preparedness. Geneva: World Health Organization (WHO). Available: <http://www.who.int/cholera/publications/>OutbreakAssessment/en/. Accessed October 15, 2016.
