# Supplementary figures and images for: Dynamics of cholera epidemics from Benin to Mauritania
Source: PLoS Negl Trop Dis. 2018 Apr 9;12(4):e0006379. doi: 10.1371/journal.pntd.0006379 (PMC5908202; doi:10.1371/journal.pntd.0006379)

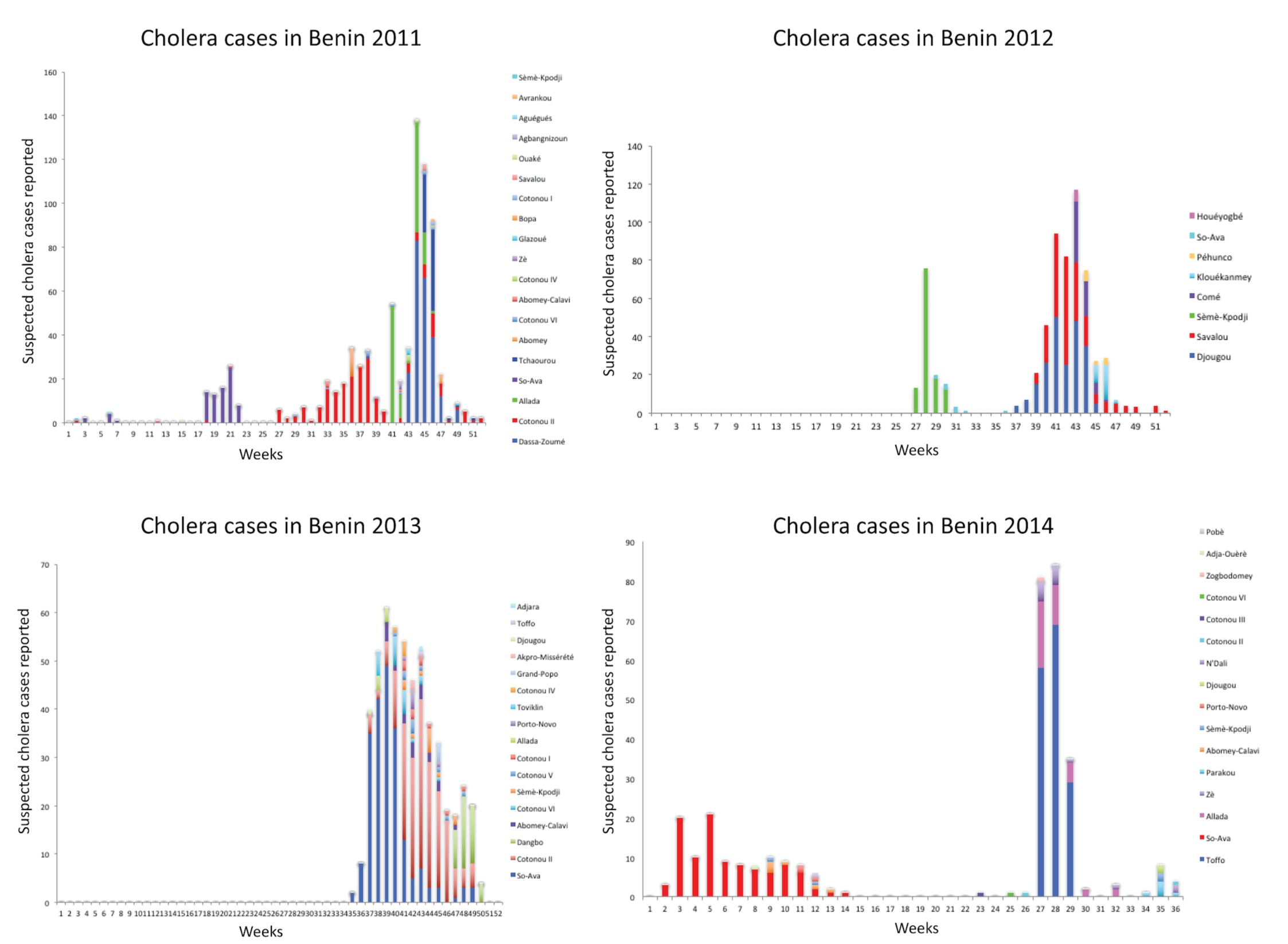

Supplement: S1 Fig — The suspected cholera cases reported are indicated on the Y-axis and the weeks are indicated on the X-axis. The communes declaring cholera cases are indicated to the right of the corresponding histogram. (TIF) [file pntd.0006379.s002.tif]

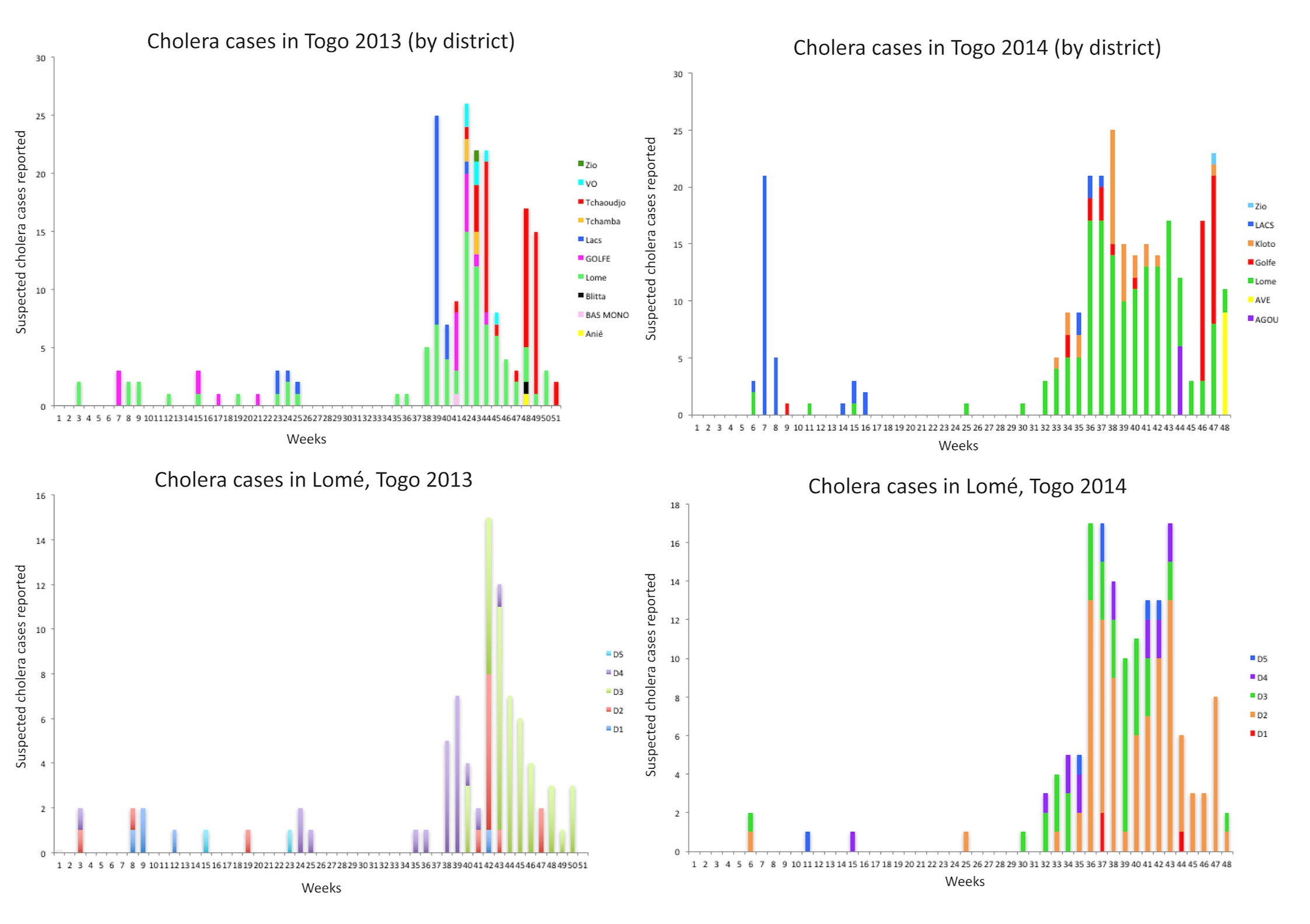

Supplement: S2 Fig — The top two histograms show weekly cholera cases by district in 2013 and 2014. The upper two histograms weekly cholera cases in Lomé in 2013 and 2014. Suspected cholera cases reported are indicated on the Y-axis and the weeks are indicated on the X-axis. The districts (upper histograms) and arrondissements of Lomé (lower histograms) declaring cholera cases are indicated to the right of the corresponding histogram. In 2012 only 61 cases were reported in Togo: 49 cases were reported in Lacs, 9 cholera cases came from Lomé (7 of which came from D2), and 3 cases were reported in Golfe. Only four cholera cases were reported in Togo in 2011. (TIF) [file pntd.0006379.s003.tif]

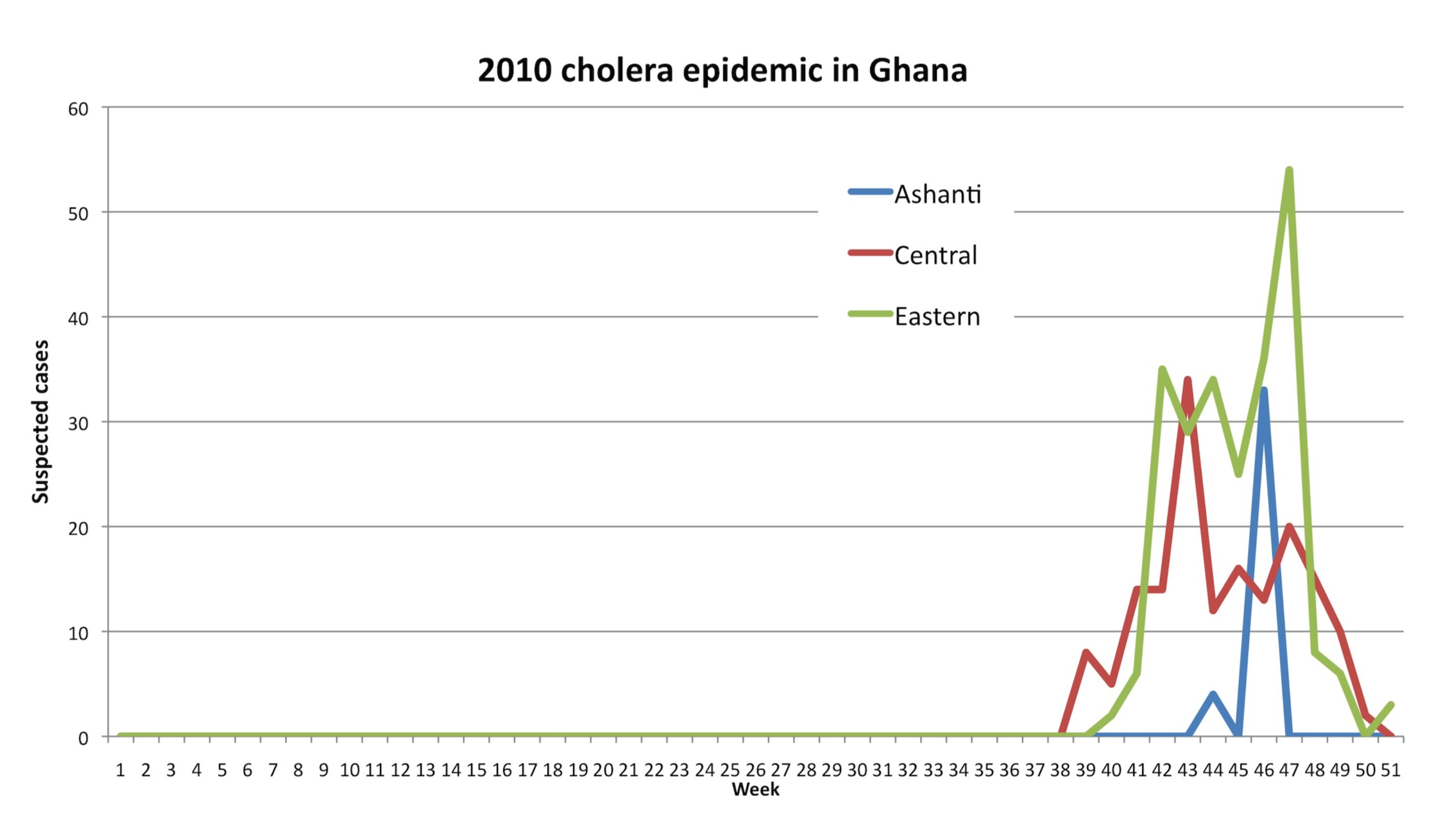

Supplement: S3 Fig — Weekly cholera cases reported in Eastern, Central, and Ashanti Region are shown. The suspected cholera cases reported are indicated on the Y-axis and the weeks are indicated on the X-axis. (TIF) [file pntd.0006379.s004.tif]

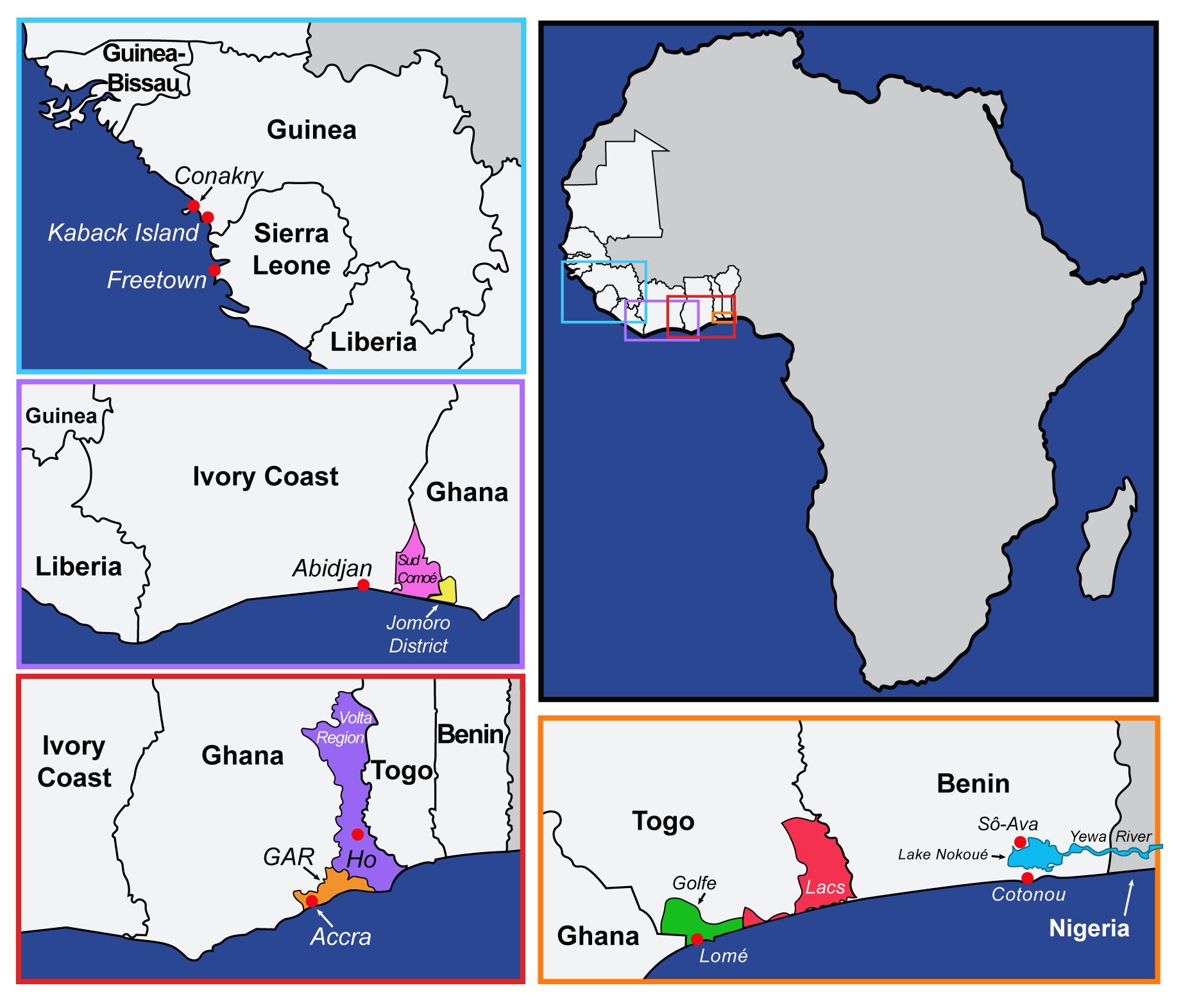

Supplement: S4 Fig — A zoom on the Guinea/Sierra Leone region (blue), southern Ivory Coast (purple), southern Ghana (red), and southern Togo and Benin (orange) are shown. In the Ivory Coast box, Sud Comoé (Ivory Coast) is indicated in pink and Jomoro District (Ghana) is indicated in yellow. In the Ghana box, Volta Region is indicated in purple and GAR (Greater Accra Region) is indicated in orange. In the Togo/Benin box, Lacs is indicated in red and Golfe is indicated in green. (TIF) [file pntd.0006379.s005.tif]

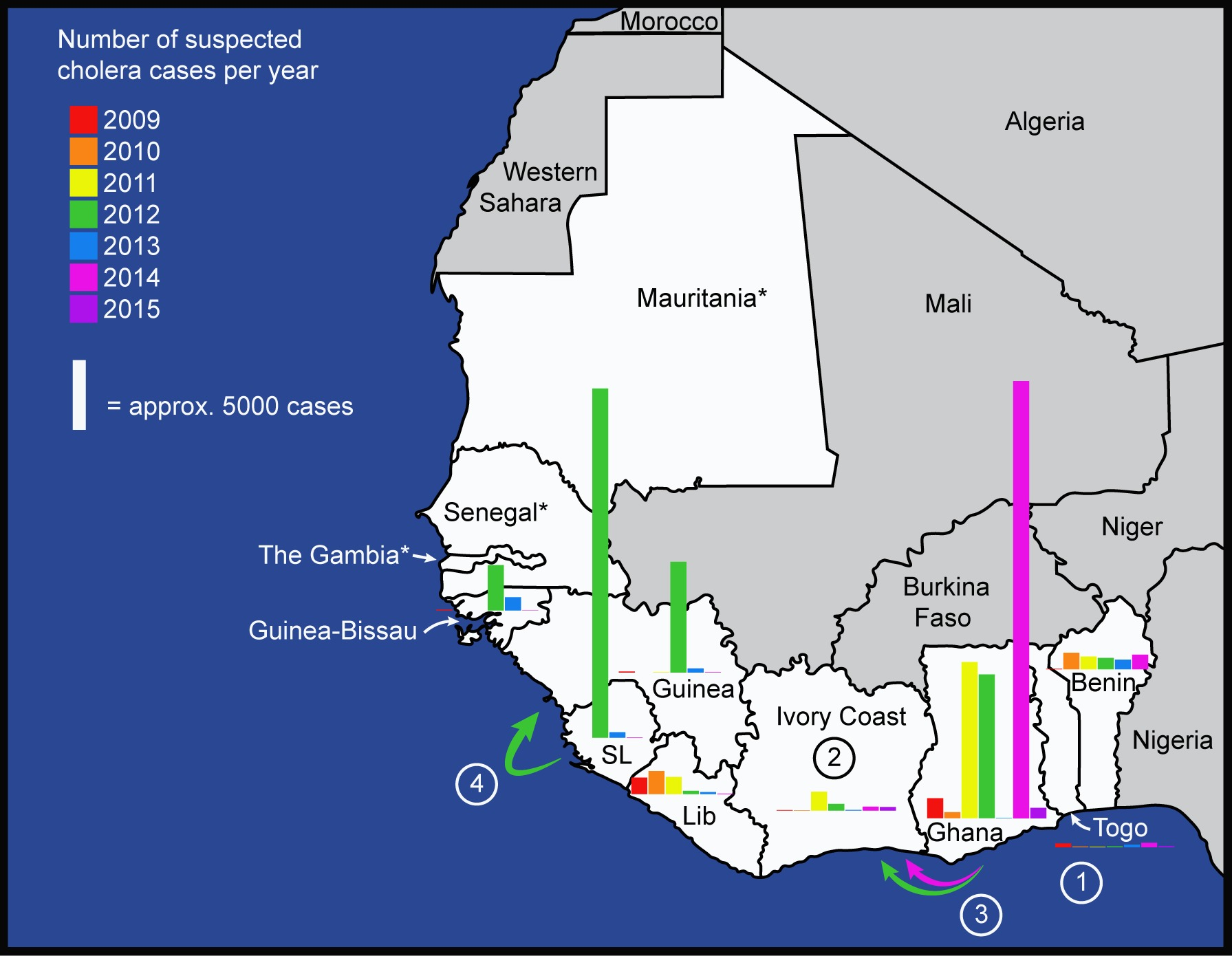

Supplement: S5 Fig — Key events and observations involved in cholera epidemics: (1) events of V. cholerae importation between Benin, Togo, and Ghana; (2) the 2011 cholera epidemic in Ivory Coast broke out following the post-election crisis and public health breakdown; (3) in Ivory Coast, 2012 and 2104 index cases had traveled from Ghana; and (4) a V. cholerae clone was imported to Guinea by a fisherman from Sierra Leone (index case of the 2012 epidemic in Guinea). Countries reporting less than 50 cases for the study period are indicated with an asterisk. Abbreviations: SL, Sierra Leone; Lib, Liberia. (TIF) [file pntd.0006379.s006.tif]
