# Supplementary material for: Dynamics of cholera epidemics from Benin to Mauritania
Source: PLoS Negl Trop Dis. 2018 Apr 9;12(4):e0006379. doi: 10.1371/journal.pntd.0006379 (PMC5908202; doi:10.1371/journal.pntd.0006379)
Supplement: S1 Table — The number of isolates corresponding to each MLVA type is indicated on the right. The environmental isolates are indicated with an asterisk (MLVA type column). (DOCX) [file pntd.0006379.s007.docx]

**S1 Table**

| **Isolate IDs** | **Epidemic**  **population** | **MLVA type** | **Locus** | | | | | | **Num of isolates** |
| --- | --- | --- | --- | --- | --- | --- | --- | --- | --- |
|  |  |  | **VC1** | **VC4** | **VC5** | **VC9** | **LAV6** | **VCMS12** |  |
| L247 | Guinea_2012 | 1* | 0 | 0 | 185 | 188 | 281 | 0 | 1 |
| G20-10 | Ghana_2010 | 2 | 179 | 232 | 188 | 188 | 269 | 272 | 1 |
| L387, L388, L390, L399, L400, G472-11, G470-11 | Togo_2010, Togo_2011, Ghana_2011 | 3 | 179 | 243 | 188 | 188 | 251 | 272 | 7 |
| G55-12 | Ghana_2012 | 4 | 179 | 243 | 188 | 188 | 263 | 272 | 1 |
| G28-11, G30-11, G704-11, G870-11, G15-10, G16-10, G17-10, G21-10, G25-10, G35-10, G13-10, G695-11, G14-10 | Ghana_2010, Ghana_2011 | 5 | 179 | 243 | 188 | 188 | 269 | 272 | 13 |
| G13-11, G60-11, G69-11, G427-11, G435-11, G68-11, G693-11, G699-11, G726-11, G781-11, G784-11, G800-11, G802-11, G807-11, G813-11, G869-11, G691-11, G18-10, G27-11, G33-11, G48-11, G50-11, G64-11, G760-11, G812-11 | Ghana_2010, Ghana_2011 | 6 | 179 | 243 | 188 | 188 | 281 | 272 | 25 |
| G762-11, G694-11, G714-11, G725-11, G732-11, G740-11, G860-11 | Ghana_2011 | 7 | 179 | 243 | 188 | 188 | 287 | 272 | 7 |
| G787-11 | Ghana_2011 | 8 | 179 | 243 | 188 | 188 | 293 | 272 | 1 |
| G56-12, G57-12, G755-11 | Ghana_2011, Ghana_2012 | 9 | 179 | 249 | 188 | 188 | 203 | 265 | 3 |
| G58-12, G54-12 | Ghana_2012 | 10 | 179 | 249 | 188 | 188 | 263 | 272 | 2 |
| G19-10 | Ghana_2010 | 11 | 179 | 249 | 188 | 188 | 269 | 272 | 1 |
| G810-11 | Ghana_2011 | 12 | 179 | 249 | 188 | 188 | 281 | 272 | 1 |
| G880-11, G867-11 | Ghana_2011 | 13 | 179 | 255 | 188 | 188 | 203 | 265 | 2 |
| G478-11 | Ghana_2011 | 14 | 179 | 255 | 188 | 188 | 281 | 272 | 1 |
| G689-11, G722-11, G820-11, G823-11, G719-11, G712-11, G816-11 | Ghana_2011 | 15 | 179 | 266 | 188 | 195 | 281 | 272 | 7 |
| L386, L389, L391, L392, L393, L394, L395, L398, L401, L402, L403, L405, L406 | Togo_2010, Togo_2011 | 16 | 185 | 220 | 188 | 160 | 233 | 272 | 13 |
| SL25 | SierraL_2012 | 17 | 185 | 238 | 188 | 188 | 299 | 272 | 1 |
| L220, L222, L227, L241 | Guinea_2012 | 18 | 185 | 243 | 188 | 181 | 287 | 272 | 4 |
| L235, L237 | Guinea_2012 | 19 | 185 | 243 | 188 | 188 | 269 | 272 | 2 |
| L234 | Guinea_2012 | 20 | 185 | 243 | 188 | 188 | 275 | 272 | 1 |
| L211, L212, L214, L215, L217, L218, L219, L236, L239, L242, L245, SL26, L219 | Guinea_2012, SierraLeone_2012 | 21 | 185 | 243 | 188 | 188 | 281 | 272 | 13 |
| L230, L231, SL30, SL32, SL34, SL36, SL37, SL38 | Guinea_2012, SierraLeone_2012 | 22 | 185 | 243 | 188 | 188 | 287 | 272 | 8 |
| SL41 | SierraL_2012 | 23 | 185 | 243 | 188 | 188 | 299 | 272 | 1 |
| L213 | Guinea_2012 | 24 | 185 | 243 | 188 | 188 | 311 | 272 | 1 |
| L225, L226 | Guinea_2012 | 25 | 185 | 243 | 188 | 195 | 281 | 272 | 2 |
| L216, L244 | Guinea_2012 | 26 | 185 | 249 | 188 | 188 | 281 | 272 | 2 |
| L223 | Guinea_2012 | 27 | 185 | 255 | 188 | 188 | 275 | 272 | 1 |
| L221, L228, L229, L233, L238, L240, L243 | Guinea_2012 | 28 | 185 | 255 | 188 | 188 | 281 | 272 | 7 |
| L224 | Guinea_2012 | 29 | 185 | 272 | 188 | 188 | 281 | 272 | 1 |
| G468-14, G372-14 | Ghana_2014 | 30 | 191 | 209 | 188 | 160 | 269 | 272 | 2 |
| G173-14, G169-14, G182-14, G487-14, | Ghana_2014 | 31 | 191 | 215 | 188 | 160 | 215 | 272 | 4 |
| G281-14 | Ghana_2014 | 32 | 191 | 215 | 188 | 160 | 269 | 265 | 1 |
| G360-14, G180-14, G262-14, G269-14, G19-14, G30-14, G36-14, G40-14, G55-14, G41-14, G93-14, G56-14, G59-14, G96-14, G114-14, G115-14, G131-14, G134-14, G135-14, G152-14, G154-14, G161-14, G204-14, G214-14, G224-14, G226-14, G239-14, G242-14, G273-14, G274-14, G278-14, G280-14, G282-14, G283-14, G286-14, G291-14, G292-14, G293-14, G297-14, G299-14, G303-14, G307-14, G309-14, G319-14, G322-14, G350-14, G381-14, G386-14, G393-14, G401-14, G408-14, G411-14, G413-14, G414-14, G455-14, G456-14, G471-14, G490-14, G494-14, G498-14, G34-14, G39-14, G171-14, G234-14, G312-14, G316-14, G330-14, G359-14, G403-14, G493-14 | Ghana_2014 | 33 | 191 | 215 | 188 | 160 | 269 | 272 | 70 |
| G271-14 | Ghana_2014 | 34 | 191 | 215 | 188 | 160 | 275 | 272 | 1 |
| L396, L397, L404, L407 | Togo_2010, Togo_2011 | 35 | 191 | 232 | 188 | 160 | 233 | 272 | 4 |
| L412, L413, L415, L416, L417, L419, L420, L421, L422 | Togo_2012 | 36 | 191 | 238 | 188 | 160 | 275 | 272 | 9 |
| L409 | Togo_2012 | 37 | 191 | 243 | 188 | 160 | 251 | 272 | 1 |
| L408 | Togo_2011 | 38 | 191 | 243 | 188 | 160 | 257 | 272 | 1 |
| L410, G10-12, G201-12, G426-12 | Togo_2012, Ghana_2012 | 39 | 191 | 266 | 188 | 160 | 239 | 272 | 4 |
| G9-12, G462-12, G463-12, G471-12, G473-12, G475-12, G477-12, G478-12, G480-12, G481-12, G483-12, G487-12, G490-12, G491-12, G496-12, G498-12, G499-12, G520-12, G521-12, G525-12, G537-12, G543-12, G517-12 | Ghana_2012 | 40 | 191 | 272 | 188 | 160 | 239 | 272 | 23 |
| L418 | Togo_2012 | 41 | 191 | 272 | 188 | 160 | 245 | 272 | 1 |
| G146-14 | Ghana_2014 | 42 | 197 | 215 | 188 | 160 | 269 | 272 | 1 |
| G160-14 | Ghana_2014 | 43 | 209 | 238 | 170 | 181 | 0 | 0 | 1 |
| L246 | Guinea_2012 | 44* | 209 | 278 | 197 | 202 | 0 | 0 | 1 |
| S5091c, S5151, S5161 | Senegal_2011 | 45 | 191 | 238 | 188 | 160 | 257 | 272 | 3 |
